# Synthesis by deamination reaction and crystal structure at 120 K of (16*Z*,19*E*)-18-oxo-*N*-(pyridin-2-yl)-6,7,9,10-tetra­hydro-18*H*-dibenzo[*h*,*o*][1,4,7]trioxa­cyclo­hexa­decine-17-carboxamide

**DOI:** 10.1107/S2056989020010968

**Published:** 2020-08-14

**Authors:** Ayalew T. Wodajo, Thi Thanh Van Tran, Hong Hieu Truong, Alexander G. Tskhovrebov, The Duan Le, Victor N. Khrustalev, Tuan Anh Le

**Affiliations:** a College of Natural and Computational Sciences, University of Gondar, 196 Gondar, Ethiopia; b Faculty of Chemistry, VNU University of Science, Vietnam National University, Hanoi, 334 Nguyen Trai, Thanh Xuan, Hanoi, Vietnam; c Inorganic Chemistry Department, Peoples’ Friendship University of Russia (RUDN University), 6 Miklukho-Maklay St., Moscow 117198, Russian Federation; d N. N. Semenov Federal Research Center, for Chemical Physics, Russian Academy of Sciences, Ul. Kosygina 4, Moscow, Russian Federation; e N. D. Zelinsky Institute of Organic Chemistry, Russian Academy of Sciences, 47 Leninsky Prosp., Moscow 119991, Russian Federation

**Keywords:** deamination reaction, aza-crown ether, dibenzo-16-crown-3, crystal structure, γ-piperidone, hydrogen bonds, C—H⋯π contacts

## Abstract

The title compound, C_27_H_24_N_2_O_5_, is a product of the deamination reaction from aza-14-crown-4 ether containing the γ-piperidone subunit. The title mol­ecule contains a 16-membered macrocycle with the conformation of the C—O—C—C—O—C—C—O—C polyether chain being *t*–*g* ^(-)^–*t*–*t*–*g* ^(+)^–*t* (*t* = *trans*, 180°; *g* = *gauche*, ±60°). The dihedral angle between the planes of the benzene rings fused to the aza-14-crown-4-ether moiety is 31.11 (14)°. The cavity size inside the macrocycle is 4.72 Å. The macrocycle is significantly flattened as a result of the extended conjugated system. Steric repulsion between the pyridyl­carboxamide fragment and the benzene ring results in a slight deviation of macrocycle from planarity. The structure also features intra­molecular hydrogen bonding, which results in a deviation of the angle between the planes of amide and pyridyl groups from planarity: this angle is 16.32 (18)°. In the crystal, the mol­ecules are linked into infinite zigzag chains *via* inter­molecular C—H⋯π contacts. The chains are bound into layers parallel to (100) by weak inter­molecular C—H⋯O hydrogen bonds.

## Abstract 2

The new title aza-crown ether has been synthesized by a deamination reaction and its crystal structure has been determined at 120 K. The title mol­ecule contains a 16-membered macrocycle with the conformation of the C—O—C—C—O—C—C—O—C polyether chain being *t*–*g* ^(-)^–*t*–*t*–*g* ^(+)^–*t* (*t* = *trans*, 180°; *g* = *gauche*, ±60°).

## Chemical context   

Nowadays, aza-crown ethers are designed, synthesized and applied as macrocyclic ligands for coordination chemistry (Hiraoka, 1982 ▸; Pedersen, 1988 ▸; Gokel & Murillo, 1996 ▸; Bradshaw & Izatt, 1997 ▸; Kolyadina *et al.*, 2013 ▸; Mazur *et al.*, 2010 ▸) and as potential anti­cancer agents with a high cytotoxicity (Anh *et al.*, 2014 ▸; Le *et al.*, 2015 ▸, 2018 ▸, 2019 ▸; Dao *et al.*, 2019 ▸). Over the last several years, new aza-crown ethers containing heterocyclic subunits such as piperidine (Levov *et al.*, 2006 ▸, 2008*a* ▸,*b* ▸; Anh *et al.*, 2008 ▸, 2012*a* ▸,*b* ▸,*c* ▸; Hieu *et al.*, 2012*a* ▸,*b* ▸, 2013 ▸), perhydro­pyrimidine (Hieu *et al.*, 2011 ▸), perhydro­triazine (Khieu *et al.*, 2011 ▸), pyridine (Anh *et al.*, 2014 ▸; Le *et al.*, 2015 ▸) and bis­pyridine (Komarova *et al.*, 2008 ▸; Sokol *et al.*, 2011 ▸) have been synthesized.

In a recent study, we condensed a γ-piperidone-containing aza-crown ether with α-amino­pyridine in the aprotic solvent *o*-xylene, which allows deamination to occur (Volkov *et al.*, 2007 ▸). After prolonged heating (5 h), the title compound was obtained in a yield of 40%. The reversible deamination reaction is apparently the result of thermodynamic control.
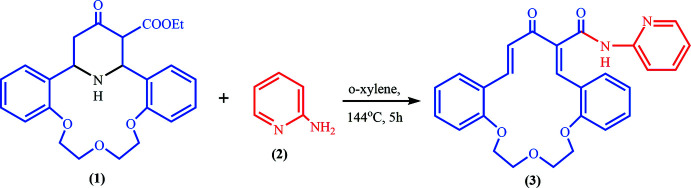


According to the *PASS* program (Filimonov *et al.*, 2014 ▸), which gives a computer prediction of biological activities, the title compound is expected to exhibit anti­allergic (72% probability) and anti­asthmatic (67%) properties, as well as to be a membrane permeability inhibitor (65%). In addition, this compound containing crown ether (–O—CH_2_—CH_2_—O—CH_2_—CH_2_—O–) and dienon fragments [–CH=CH—C(O)—CH=CH–] could act as a good ligand in coordination chemistry.

## Structural commentary   

**Figure 1:**
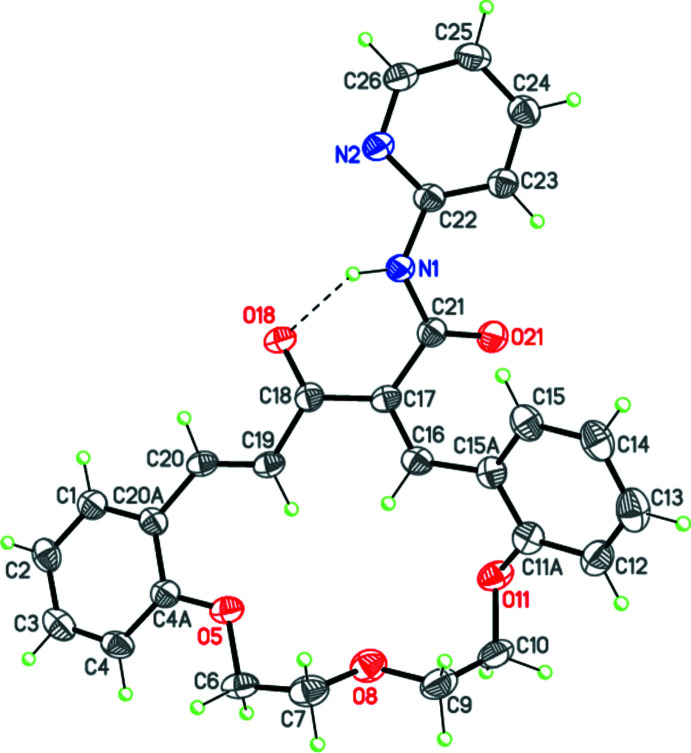
Mol­ecular structure of (**3**) with displacement ellipsoids shown at the 50% probability level. H atoms are presented as small spheres of arbitrary radius. Dashed line indicates the intra­molecular N—H⋯O hydrogen bond.

The title compound, (**3**), is a product of the deamination reaction starting from aza-14-crown-4 ether containing the γ-piperidone subunit (**1**). The mol­ecular structure of (**3**) is presented in Fig. 1 ▸. The mol­ecule contains a 16-membered macrocycle with the C7–O8–C9–C10–O11–C12–C13–O14–C15 polyether chain exhibiting a *t*–*g* ^(-)^–*t*–*t*–*g* ^(+)^–*t* (*t* = trans, 180°; *g* = gauche, ±60°) conformation. The dihedral angle between the mean planes of the benzene rings fused to the aza-14-crown-4-ether moiety is 31.11 (14)°. The cavity size inside the macrocycle, determined as a double-mean distance between the C16, C19, O5, O8 and O11 atoms and the center of this penta­gon, is 4.72 Å. The macrocycle is significantly flattened because of the extended conjugated system. The steric repulsion between the 17-pyridyl­carboxamide fragment and the aromatic ring (C11*A*/C12–C15/C15*A*) results in a slight deviation of the macrocycle from planarity. The mol­ecular structure also features intra­molecular hydrogen bonds (Table 1 ▸), which result in the deviation of the amide and pyridyl groups from coplanarity, the angle between their main planes being 16.32 (18)°. In addition, the intra­molecular N1—H1*N*⋯O18 hydrogen bond has a significant impact on the structure, preventing the C11*A*/C12–C15/C15*A* benzene ring from being conjugated with the C16=C17 double bond.

## Supra­molecular features   

**Figure 2:**
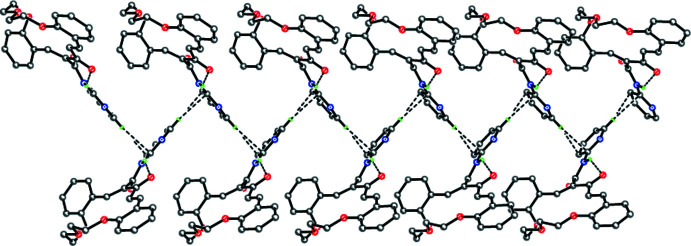
The chain of mol­ecules of (**3**) along the *c* axis. Dashed lines indicate the intra­molecular N—H⋯O hydrogen bonds and the inter­molecular C—H⋯π contacts.

**Figure 3:**
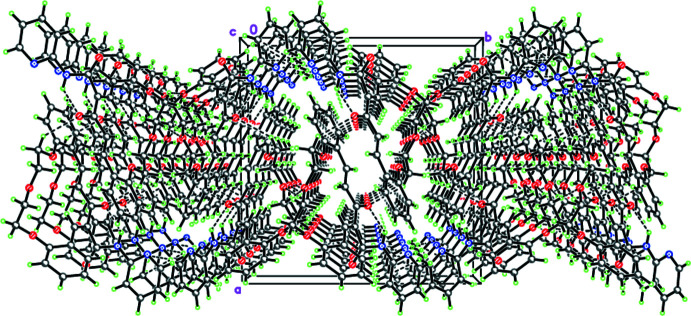
Crystal packing of (**3)** illustrating the two-tier puckered layer parallel to (100). Dashed lines indicate the intra­molecular N—H⋯O and the inter­molecular C—H⋯π and C—H⋯O hydrogen bonds.

In the crystal, mol­ecules of (**3**) are linked into infinite zigzag chains *via* inter­molecular C26—H⋯π(C22) contacts (Fig. 2 ▸). A similar supra­molecular motif was previously observed by our group (Tskhovrebov *et al.*, 2019 ▸; Repina *et al.*, 2020 ▸). The chains are linked into two-tier puckered layers parallel to (100) by weak inter­molecular C—H⋯O hydrogen bonds (Table 1 ▸, Fig. 3 ▸).

## Database survey   

A search of the Cambridge Structural Database (CSD version 5.41, update of March 2020; Groom *et al.*, 2016 ▸) revealed the existence of several structurally similar compounds. Since members of our group reported the synthesis of dibenzopiperazidino­aza-14-crown-4 for the first time (Levov *et al.*, 2006 ▸), several relevant macrocyclic crown ethers have been prepared and structurally characterized (Hieu *et al.*, 2012*a* ▸, 2016 ▸; Polyakova *et al.*, 2016 ▸, 2018 ▸; Sokol *et al.*, 2014 ▸; Nguyen *et al.*, 2017 ▸; Anh *et al.*, 2012*c* ▸ and references therein). The aforementioned macrocyclic crown ethers contain an O_3_C_4_ linear chain fragment appended to the two aryl rings. The O atoms in the macrocycles appear to be in an *sp* ^3^-hybridized state with C—O—C angles close to 120°. Overall, the metrical parameters in this type of macrocyclic ligand are not remarkable.

## Synthesis and crystallization   

Aza-crown ether (**1**) was synthesized according to the procedure described previously (Levov *et al.*, 2008*a* ▸), and purified by recrystallization from ethanol. α-Amino­pyridine and *o*-xylene were acquired from Aldrich. All solvents were HPLC grade and used without any further purification.

A solution of 2.0 g (4.7 mmol) aza-crown ether (**1**) and 0.44 g (4.7 mmol) α-amino­pyridine (**2**) in 10 ml *o*-xylene was refluxed with stirring for 5 h (monitored by TLC until the disappearance of the starting organic compound spots). The solvent was evaporated under vacuum, then the residue was purified by column chromatography (ethyl acetate:*n*-hexane = 5:1) and recrystallized from ethanol to obtain 1.27 g of pure compound (**3**) as single crystals in 58% yield. T_mlt_ = 482–484 K. *R*_f_ = 0.66 (ethyl acetate, silufol). IR, *ν*, cm^−1^: 1687 (C=O), 1638 (HN—C=O), 3317 (NH). ^1^H NMR (CDCl_3_, 400 MHz, 300 K): 3.89 (*m*, 2H, *J* = 4.3 and 2.0 Hz, CH_2_OCH_2_), 4.01 (*m*, 2H, *J* = 4.3 and 1.7 Hz, CH_2_OCH_2_), 4.33 (*m*, 4H, Ph–O–CH_2_), 6.91–7.75 (*m*, 11H, H_ar­yl_, H_pyridine_), 7.73 (*d*, 1H, *J* = 15.7 Hz, H18), 8.18 (*d*, 1H, *J* = 15.7 Hz, H17), 8.32 (*d*, 1H, *J* = 5.4 Hz, H_pyridine_), 8.36 (*s*, 1H, H14). Mass spectrum, *m*/*z* (*I*_max_, %): 456 [*M*]^+^ (4), 428 (1), 309 (4), 283 (3), 265 (3), 238 (25), 221 (18), 210 (50), 189 (10), 173 (89), 159 (20), 147 (38), 131 (100), 118 (48), 115 (51), 103 (27), 91 (81), 89 (52), 78 (65), 45 (38). Analysis calculated for C_27_H_24_N_2_O_5_, %: C, 71.04; H, 5.30; N, 6.14. Found: C, 70.82; H, 5.34; N, 6.01.

## Refinement   

Crystal data, data collection and structure refinement details are summarized in Table 2 ▸. The hydrogen atom of the amino group was localized in a difference-Fourier map and refined isotropically with fixed displacement parameters [*U*_iso_(H) = 1.2*U*_eq_(N)]. The other hydrogen atoms were placed in calculated positions with C—H = 0.95–0.99 Å and refined as riding with fixed isotropic displacement parameters [*U*_iso_(H) = 1.2*U*_eq_(C)].

## Supplementary Material

Crystal structure: contains datablock(s) I. DOI: 10.1107/S2056989020010968/yk2136sup1.cif

Structure factors: contains datablock(s) I. DOI: 10.1107/S2056989020010968/yk2136Isup2.hkl

CCDC reference: 2022314

Additional supporting information:  crystallographic information; 3D view; checkCIF report

## Figures and Tables

**Table 1:** Hydrogen-bond geometry (Å, °)

*D*—H⋯*A*	*D*—H	H⋯*A*	*D*⋯*A*	*D*—H⋯*A*
N1—H1*N*⋯O18	0.88 (3)	2.04 (3)	2.737 (3)	135 (3)
C19—H19⋯O5	0.95	2.22	2.823 (4)	120
C23—H23⋯O21	0.95	2.34	2.903 (4)	117
C6—H6*B*⋯O18^i^	0.99	2.50	3.323 (4)	140
C9—H9*A*⋯O8^ii^	0.99	2.48	3.447 (4)	165
C10—H10*A*⋯O11^ii^	0.99	2.41	3.238 (4)	140
C26—H26⋯C22^iii^	0.95	2.76	3.678 (4)	164

Symmetry codes: (i) 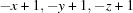; (ii) 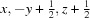; (iii) 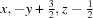.

**Table 2:** Experimental details

Crystal data	
Chemical formula	C_27_H_24_N_2_O_5_
*M*_r_	456.48
Crystal system, space group	Monoclinic, *P*2_1_/*c*
Temperature (K)	120
*a*, *b*, *c* (Å)	17.021 (6), 16.519 (5), 8.079 (3)
β (°)	97.552 (8)
*V* (Å^3^)	2251.9 (13)
*Z*	4
Radiation type	Mo *K*α
μ (mm^−1^)	0.09
Crystal size (mm)	0.20 × 0.20 × 0.05

Data collection	
Diffractometer	Bruker APEXII CCD
Absorption correction	Multi-scan (*SADABS*; Sheldrick, 2003 ▸)
*T*_min_, *T*_max_	0.975, 0.987
No. of measured, independent and observed [*I* > 2σ(*I*)] reflections	13715, 4398, 2301
*R*_int_	0.100
(sin θ/λ)_max_ (Å^−1^)	0.617

Refinement	
*R*[*F* ^2^ > 2σ(*F* ^2^)], *wR*(*F* ^2^), *S*	0.060, 0.169, 0.97
No. of reflections	4398
No. of parameters	310
H-atom treatment	H atoms treated by a mixture of independent and constrained refinement
Δρ_max_, Δρ_min_ (e Å^−3^)	0.24, −0.27

Computer programs: *APEX2* (Bruker, 2005 ▸), *SAINT* (Bruker, 2001 ▸), *SHELXT* (Sheldrick, 2015*a* ▸), *SHELXL* (Sheldrick, 2015*b* ▸) and *SHELXTL* (Sheldrick, 2015*b* ▸).

## supplementary crystallographic information

### Crystal data

**Table:**

C_27_H_24_N_2_O_5_	*F*(000) = 960
*M**_r_* = 456.48	*D*_x_ = 1.346 Mg m^−^^3^
Monoclinic, *P*2_1_/*c*	Mo *K*α radiation, λ = 0.71073 Å
*a* = 17.021 (6) Å	Cell parameters from 1174 reflections
*b* = 16.519 (5) Å	θ = 2.5–23.8°
*c* = 8.079 (3) Å	µ = 0.09 mm^−^^1^
β = 97.552 (8)°	*T* = 120 K
*V* = 2251.9 (13) Å^3^	Plate, light-yellow
*Z* = 4	0.20 × 0.20 × 0.05 mm

### Data collection

**Table:**

Bruker APEXII CCD diffractometer	2301 reflections with *I* > 2σ(*I*)
Radiation source: fine-focus sealed tube	*R*_int_ = 0.100
φ and ω scans	θ_max_ = 26.0°, θ_min_ = 2.4°
Absorption correction: multi-scan (SADABS; Sheldrick, 2003)	*h* = −14→20
*T*_min_ = 0.975, *T*_max_ = 0.987	*k* = −19→20
13715 measured reflections	*l* = −9→9
4398 independent reflections	

### Refinement

**Table:**

Refinement on *F*^2^	Primary atom site location: difference Fourier map
Least-squares matrix: full	Secondary atom site location: difference Fourier map
*R*[*F*^2^ > 2σ(*F*^2^)] = 0.060	Hydrogen site location: mixed
*wR*(*F*^2^) = 0.169	H atoms treated by a mixture of independent and constrained refinement
*S* = 0.97	*w* = 1/[σ^2^(*F*_o_^2^) + (0.0735*P*)^2^] where *P* = (*F*_o_^2^ + 2*F*_c_^2^)/3
4398 reflections	(Δ/σ)_max_ < 0.001
310 parameters	Δρ_max_ = 0.24 e Å^−^^3^
0 restraints	Δρ_min_ = −0.27 e Å^−^^3^

### Special details

**Table:**

Geometry. All esds (except the esd in the dihedral angle between two l.s. planes) are estimated using the full covariance matrix. The cell esds are taken into account individually in the estimation of esds in distances, angles and torsion angles; correlations between esds in cell parameters are only used when they are defined by crystal symmetry. An approximate (isotropic) treatment of cell esds is used for estimating esds involving l.s. planes.

### Fractional atomic coordinates and isotropic or equivalent isotropic displacement parameters (Å^2^)

**Table:**

	*x*	*y*	*z*	*U*_iso_*/*U*_eq_	
N1	0.81155 (16)	0.57786 (15)	0.3654 (4)	0.0314 (7)	
H1N	0.7620 (19)	0.5900 (19)	0.327 (4)	0.038*	
N2	0.84697 (15)	0.67951 (15)	0.1997 (4)	0.0370 (7)	
C1	0.39211 (18)	0.41274 (18)	0.1565 (4)	0.0323 (8)	
H1	0.4052	0.4436	0.0648	0.039*	
C2	0.31862 (19)	0.37494 (19)	0.1440 (5)	0.0354 (8)	
H2	0.2822	0.3795	0.0447	0.043*	
C3	0.29920 (19)	0.33067 (19)	0.2777 (5)	0.0368 (9)	
H3	0.2496	0.3037	0.2694	0.044*	
C4	0.35155 (19)	0.32526 (18)	0.4240 (5)	0.0342 (8)	
H4	0.3369	0.2958	0.5161	0.041*	
C4A	0.42529 (18)	0.36265 (17)	0.4368 (4)	0.0290 (8)	
O5	0.47971 (12)	0.35990 (12)	0.5778 (3)	0.0328 (6)	
C6	0.4577 (2)	0.31983 (18)	0.7232 (4)	0.0335 (8)	
H6A	0.4500	0.2613	0.7007	0.040*	
H6B	0.4073	0.3425	0.7517	0.040*	
C7	0.5223 (2)	0.3323 (2)	0.8653 (4)	0.0400 (9)	
H7A	0.5320	0.3909	0.8830	0.048*	
H7B	0.5060	0.3094	0.9688	0.048*	
O8	0.59254 (13)	0.29397 (13)	0.8301 (3)	0.0391 (6)	
C9	0.6507 (2)	0.2893 (2)	0.9744 (4)	0.0428 (9)	
H9A	0.6300	0.2570	1.0623	0.051*	
H9B	0.6632	0.3443	1.0189	0.051*	
C10	0.7239 (2)	0.2505 (2)	0.9269 (4)	0.0411 (9)	
H10A	0.7614	0.2398	1.0290	0.049*	
H10B	0.7099	0.1980	0.8719	0.049*	
O11	0.76185 (13)	0.30110 (12)	0.8158 (3)	0.0364 (6)	
C11A	0.80461 (18)	0.36681 (18)	0.8857 (4)	0.0304 (8)	
C12	0.85596 (19)	0.3632 (2)	1.0331 (4)	0.0386 (9)	
H12	0.8594	0.3154	1.0991	0.046*	
C13	0.9026 (2)	0.4302 (2)	1.0842 (5)	0.0440 (9)	
H13	0.9373	0.4287	1.1863	0.053*	
C14	0.89803 (19)	0.4990 (2)	0.9853 (4)	0.0389 (9)	
H14	0.9307	0.5443	1.0190	0.047*	
C15	0.84686 (18)	0.5026 (2)	0.8396 (4)	0.0358 (8)	
H15	0.8443	0.5503	0.7735	0.043*	
C15A	0.79813 (18)	0.43628 (18)	0.7869 (4)	0.0293 (8)	
C16	0.73583 (18)	0.43972 (17)	0.6437 (4)	0.0284 (7)	
H16	0.6877	0.4133	0.6588	0.034*	
C17	0.73683 (18)	0.47504 (16)	0.4934 (4)	0.0260 (7)	
C18	0.66355 (17)	0.48163 (17)	0.3709 (4)	0.0283 (8)	
O18	0.66282 (13)	0.52429 (13)	0.2450 (3)	0.0376 (6)	
C19	0.59118 (18)	0.43833 (17)	0.4033 (4)	0.0289 (8)	
H19	0.5920	0.4063	0.5011	0.035*	
C20	0.52515 (18)	0.44386 (17)	0.2969 (4)	0.0282 (7)	
H20	0.5289	0.4775	0.2028	0.034*	
C20A	0.44743 (17)	0.40665 (17)	0.3006 (4)	0.0273 (7)	
C21	0.81515 (18)	0.50452 (18)	0.4451 (4)	0.0288 (8)	
O21	0.87634 (12)	0.46652 (13)	0.4817 (3)	0.0381 (6)	
C22	0.87370 (18)	0.62108 (17)	0.3072 (4)	0.0292 (7)	
C23	0.95325 (18)	0.60770 (18)	0.3617 (4)	0.0301 (8)	
H23	0.9696	0.5666	0.4410	0.036*	
C24	1.00802 (19)	0.6561 (2)	0.2971 (4)	0.0355 (8)	
H24	1.0631	0.6481	0.3305	0.043*	
C25	0.9825 (2)	0.7161 (2)	0.1840 (5)	0.0417 (9)	
H25	1.0192	0.7501	0.1382	0.050*	
C26	0.9017 (2)	0.7254 (2)	0.1394 (5)	0.0438 (10)	
H26	0.8839	0.7666	0.0615	0.053*	

### Atomic displacement parameters (Å^2^)

**Table:**

	*U*^11^	*U*^22^	*U*^33^	*U*^12^	*U*^13^	*U*^23^
N1	0.0264 (14)	0.0261 (14)	0.0424 (18)	0.0027 (12)	0.0064 (13)	0.0087 (13)
N2	0.0330 (15)	0.0306 (15)	0.0483 (19)	0.0036 (13)	0.0081 (14)	0.0128 (14)
C1	0.0338 (18)	0.0260 (17)	0.037 (2)	0.0007 (14)	0.0067 (16)	−0.0016 (15)
C2	0.0296 (18)	0.0328 (18)	0.044 (2)	0.0021 (15)	0.0055 (16)	−0.0072 (17)
C3	0.0319 (18)	0.0277 (18)	0.053 (2)	−0.0039 (15)	0.0136 (17)	−0.0101 (17)
C4	0.0352 (19)	0.0222 (16)	0.048 (2)	−0.0020 (14)	0.0158 (17)	0.0009 (16)
C4A	0.0315 (17)	0.0189 (16)	0.038 (2)	0.0017 (14)	0.0080 (15)	0.0003 (14)
O5	0.0388 (13)	0.0274 (12)	0.0334 (14)	−0.0016 (10)	0.0091 (11)	0.0052 (10)
C6	0.046 (2)	0.0230 (16)	0.035 (2)	0.0019 (15)	0.0185 (16)	0.0036 (15)
C7	0.052 (2)	0.0347 (19)	0.036 (2)	0.0038 (17)	0.0167 (17)	0.0016 (16)
O8	0.0459 (14)	0.0396 (14)	0.0328 (14)	0.0032 (11)	0.0094 (11)	0.0011 (11)
C9	0.053 (2)	0.045 (2)	0.030 (2)	−0.0063 (18)	0.0018 (18)	0.0089 (17)
C10	0.052 (2)	0.036 (2)	0.033 (2)	−0.0056 (17)	0.0012 (17)	0.0130 (16)
O11	0.0494 (14)	0.0264 (12)	0.0326 (14)	−0.0041 (10)	0.0030 (11)	0.0038 (10)
C11A	0.0341 (18)	0.0297 (17)	0.0280 (19)	0.0026 (14)	0.0058 (15)	−0.0051 (15)
C12	0.0406 (19)	0.043 (2)	0.031 (2)	0.0058 (17)	0.0032 (16)	0.0031 (17)
C13	0.036 (2)	0.060 (2)	0.035 (2)	0.0046 (18)	0.0014 (16)	−0.012 (2)
C14	0.0308 (18)	0.045 (2)	0.040 (2)	−0.0020 (16)	0.0038 (16)	−0.0132 (18)
C15	0.0303 (17)	0.0365 (19)	0.041 (2)	0.0004 (15)	0.0061 (16)	−0.0066 (16)
C15A	0.0293 (17)	0.0279 (17)	0.0311 (19)	0.0032 (14)	0.0053 (15)	−0.0030 (15)
C16	0.0321 (17)	0.0189 (15)	0.034 (2)	−0.0030 (13)	0.0057 (14)	−0.0030 (14)
C17	0.0336 (18)	0.0142 (14)	0.0305 (19)	0.0014 (13)	0.0056 (14)	−0.0012 (13)
C18	0.0302 (17)	0.0185 (15)	0.036 (2)	0.0013 (13)	0.0022 (15)	−0.0011 (15)
O18	0.0383 (13)	0.0349 (13)	0.0389 (15)	−0.0051 (11)	0.0025 (11)	0.0145 (11)
C19	0.0360 (18)	0.0209 (16)	0.0307 (19)	−0.0001 (14)	0.0071 (15)	0.0027 (14)
C20	0.0364 (19)	0.0178 (15)	0.0311 (19)	−0.0013 (13)	0.0069 (15)	0.0017 (13)
C20A	0.0265 (16)	0.0171 (15)	0.038 (2)	−0.0023 (13)	0.0057 (15)	−0.0041 (14)
C21	0.0318 (18)	0.0236 (16)	0.0310 (19)	0.0016 (14)	0.0037 (15)	−0.0004 (14)
O21	0.0321 (13)	0.0325 (12)	0.0506 (16)	0.0076 (11)	0.0084 (11)	0.0094 (11)
C22	0.0322 (17)	0.0194 (15)	0.037 (2)	−0.0002 (14)	0.0073 (15)	−0.0020 (14)
C23	0.0319 (18)	0.0255 (16)	0.033 (2)	0.0019 (14)	0.0065 (15)	−0.0005 (15)
C24	0.0322 (18)	0.0316 (18)	0.043 (2)	−0.0005 (15)	0.0059 (16)	−0.0023 (16)
C25	0.037 (2)	0.037 (2)	0.054 (3)	−0.0060 (16)	0.0131 (17)	0.0098 (18)
C26	0.042 (2)	0.035 (2)	0.055 (3)	0.0005 (17)	0.0094 (18)	0.0177 (18)

### Geometric parameters (Å, º)

**Table:**

N1—C21	1.370 (4)	C11A—C12	1.383 (4)
N1—C22	1.407 (4)	C11A—C15A	1.394 (4)
N1—H1N	0.88 (3)	C12—C13	1.392 (5)
N2—C22	1.338 (4)	C12—H12	0.9500
N2—C26	1.341 (4)	C13—C14	1.386 (5)
C1—C2	1.390 (4)	C13—H13	0.9500
C1—C20A	1.401 (4)	C14—C15	1.371 (4)
C1—H1	0.9500	C14—H14	0.9500
C2—C3	1.380 (5)	C15—C15A	1.405 (4)
C2—H2	0.9500	C15—H15	0.9500
C3—C4	1.386 (5)	C15A—C16	1.464 (4)
C3—H3	0.9500	C16—C17	1.350 (4)
C4—C4A	1.390 (4)	C16—H16	0.9500
C4—H4	0.9500	C17—C18	1.491 (4)
C4A—O5	1.371 (4)	C17—C21	1.518 (4)
C4A—C20A	1.411 (4)	C18—O18	1.236 (4)
O5—C6	1.441 (4)	C18—C19	1.477 (4)
C6—C7	1.495 (5)	C19—C20	1.326 (4)
C6—H6A	0.9900	C19—H19	0.9500
C6—H6B	0.9900	C20—C20A	1.463 (4)
C7—O8	1.415 (4)	C20—H20	0.9500
C7—H7A	0.9900	C21—O21	1.219 (3)
C7—H7B	0.9900	C22—C23	1.385 (4)
O8—C9	1.429 (4)	C23—C24	1.381 (4)
C9—C10	1.495 (5)	C23—H23	0.9500
C9—H9A	0.9900	C24—C25	1.380 (4)
C9—H9B	0.9900	C24—H24	0.9500
C10—O11	1.440 (4)	C25—C26	1.383 (5)
C10—H10A	0.9900	C25—H25	0.9500
C10—H10B	0.9900	C26—H26	0.9500
O11—C11A	1.385 (4)		
			
C21—N1—C22	128.1 (3)	C11A—C12—H12	120.3
C21—N1—H1N	110 (2)	C13—C12—H12	120.3
C22—N1—H1N	120 (2)	C14—C13—C12	119.7 (3)
C22—N2—C26	116.8 (3)	C14—C13—H13	120.2
C2—C1—C20A	121.9 (3)	C12—C13—H13	120.2
C2—C1—H1	119.1	C15—C14—C13	120.7 (3)
C20A—C1—H1	119.1	C15—C14—H14	119.6
C3—C2—C1	119.2 (3)	C13—C14—H14	119.6
C3—C2—H2	120.4	C14—C15—C15A	120.7 (3)
C1—C2—H2	120.4	C14—C15—H15	119.7
C2—C3—C4	120.5 (3)	C15A—C15—H15	119.7
C2—C3—H3	119.7	C11A—C15A—C15	117.9 (3)
C4—C3—H3	119.7	C11A—C15A—C16	118.6 (3)
C3—C4—C4A	120.5 (3)	C15—C15A—C16	123.1 (3)
C3—C4—H4	119.7	C17—C16—C15A	129.4 (3)
C4A—C4—H4	119.7	C17—C16—H16	115.3
O5—C4A—C4	123.5 (3)	C15A—C16—H16	115.3
O5—C4A—C20A	116.4 (3)	C16—C17—C18	121.4 (3)
C4—C4A—C20A	120.1 (3)	C16—C17—C21	119.0 (3)
C4A—O5—C6	118.2 (2)	C18—C17—C21	119.5 (3)
O5—C6—C7	108.6 (3)	O18—C18—C19	120.2 (3)
O5—C6—H6A	110.0	O18—C18—C17	120.3 (3)
C7—C6—H6A	110.0	C19—C18—C17	119.4 (3)
O5—C6—H6B	110.0	C20—C19—C18	120.5 (3)
C7—C6—H6B	110.0	C20—C19—H19	119.8
H6A—C6—H6B	108.3	C18—C19—H19	119.8
O8—C7—C6	109.9 (3)	C19—C20—C20A	130.5 (3)
O8—C7—H7A	109.7	C19—C20—H20	114.7
C6—C7—H7A	109.7	C20A—C20—H20	114.7
O8—C7—H7B	109.7	C1—C20A—C4A	117.8 (3)
C6—C7—H7B	109.7	C1—C20A—C20	117.6 (3)
H7A—C7—H7B	108.2	C4A—C20A—C20	124.6 (3)
C7—O8—C9	112.0 (3)	O21—C21—N1	123.6 (3)
O8—C9—C10	109.0 (3)	O21—C21—C17	121.6 (3)
O8—C9—H9A	109.9	N1—C21—C17	114.7 (3)
C10—C9—H9A	109.9	N2—C22—C23	123.8 (3)
O8—C9—H9B	109.9	N2—C22—N1	112.1 (3)
C10—C9—H9B	109.9	C23—C22—N1	124.0 (3)
H9A—C9—H9B	108.3	C24—C23—C22	117.9 (3)
O11—C10—C9	111.6 (3)	C24—C23—H23	121.1
O11—C10—H10A	109.3	C22—C23—H23	121.1
C9—C10—H10A	109.3	C25—C24—C23	119.8 (3)
O11—C10—H10B	109.3	C25—C24—H24	120.1
C9—C10—H10B	109.3	C23—C24—H24	120.1
H10A—C10—H10B	108.0	C24—C25—C26	117.9 (3)
C11A—O11—C10	117.1 (3)	C24—C25—H25	121.1
C12—C11A—O11	123.8 (3)	C26—C25—H25	121.1
C12—C11A—C15A	121.5 (3)	N2—C26—C25	123.9 (3)
O11—C11A—C15A	114.4 (3)	N2—C26—H26	118.1
C11A—C12—C13	119.5 (3)	C25—C26—H26	118.1
			
C20A—C1—C2—C3	0.5 (5)	C21—C17—C18—O18	−14.0 (4)
C1—C2—C3—C4	1.3 (5)	C16—C17—C18—C19	−9.2 (4)
C2—C3—C4—C4A	−1.7 (5)	C21—C17—C18—C19	167.5 (3)
C3—C4—C4A—O5	179.8 (3)	O18—C18—C19—C20	0.9 (5)
C3—C4—C4A—C20A	0.1 (5)	C17—C18—C19—C20	179.4 (3)
C4—C4A—O5—C6	−3.5 (4)	C18—C19—C20—C20A	179.3 (3)
C20A—C4A—O5—C6	176.3 (2)	C2—C1—C20A—C4A	−2.0 (5)
C4A—O5—C6—C7	−173.9 (3)	C2—C1—C20A—C20	175.8 (3)
O5—C6—C7—O8	−64.4 (3)	O5—C4A—C20A—C1	−178.0 (3)
C6—C7—O8—C9	−167.7 (3)	C4—C4A—C20A—C1	1.7 (4)
C7—O8—C9—C10	−178.4 (3)	O5—C4A—C20A—C20	4.3 (4)
O8—C9—C10—O11	67.3 (3)	C4—C4A—C20A—C20	−175.9 (3)
C9—C10—O11—C11A	76.2 (3)	C19—C20—C20A—C1	−169.6 (3)
C10—O11—C11A—C12	44.2 (4)	C19—C20—C20A—C4A	8.0 (5)
C10—O11—C11A—C15A	−142.0 (3)	C22—N1—C21—O21	2.3 (5)
O11—C11A—C12—C13	173.2 (3)	C22—N1—C21—C17	179.3 (3)
C15A—C11A—C12—C13	−0.2 (5)	C16—C17—C21—O21	38.3 (4)
C11A—C12—C13—C14	−1.2 (5)	C18—C17—C21—O21	−138.4 (3)
C12—C13—C14—C15	1.5 (5)	C16—C17—C21—N1	−138.8 (3)
C13—C14—C15—C15A	−0.3 (5)	C18—C17—C21—N1	44.4 (4)
C12—C11A—C15A—C15	1.4 (5)	C26—N2—C22—C23	2.0 (5)
O11—C11A—C15A—C15	−172.7 (3)	C26—N2—C22—N1	178.7 (3)
C12—C11A—C15A—C16	−172.5 (3)	C21—N1—C22—N2	164.2 (3)
O11—C11A—C15A—C16	13.5 (4)	C21—N1—C22—C23	−19.1 (5)
C14—C15—C15A—C11A	−1.1 (5)	N2—C22—C23—C24	−2.0 (5)
C14—C15—C15A—C16	172.4 (3)	N1—C22—C23—C24	−178.3 (3)
C11A—C15A—C16—C17	−145.3 (3)	C22—C23—C24—C25	0.9 (5)
C15—C15A—C16—C17	41.2 (5)	C23—C24—C25—C26	0.0 (5)
C15A—C16—C17—C18	−171.7 (3)	C22—N2—C26—C25	−1.0 (5)
C15A—C16—C17—C21	11.6 (5)	C24—C25—C26—N2	0.1 (6)
C16—C17—C18—O18	169.3 (3)		

### Hydrogen-bond geometry (Å, º)

**Table:**

*D*—H···*A*	*D*—H	H···*A*	*D*···*A*	*D*—H···*A*
N1—H1*N*···O18	0.88 (3)	2.04 (3)	2.737 (3)	135 (3)
C19—H19···O5	0.95	2.22	2.823 (4)	120
C23—H23···O21	0.95	2.34	2.903 (4)	117
C6—H6*B*···O18^i^	0.99	2.50	3.323 (4)	140
C9—H9*A*···O8^ii^	0.99	2.48	3.447 (4)	165
C10—H10*A*···O11^ii^	0.99	2.41	3.238 (4)	140
C26—H26···C22^iii^	0.95	2.76	3.678 (4)	164

Symmetry codes: (i) −*x*+1, −*y*+1, −*z*+1; (ii) *x*, −*y*+1/2, *z*+1/2; (iii) *x*, −*y*+3/2, *z*−1/2.
